# An Fc-Optimized CD133 Antibody for Induction of NK Cell Reactivity against B Cell Acute Lymphoblastic Leukemia

**DOI:** 10.3390/cancers13071632

**Published:** 2021-04-01

**Authors:** Fabian Riegg, Martina S. Lutz, Bastian J. Schmied, Jonas S. Heitmann, Manon Queudeville, Peter Lang, Gundram Jung, Helmut R. Salih, Melanie Märklin

**Affiliations:** 1 Clinical Collaboration Unit Translational Immunology, German Cancer Consortium (DKTK), Department of Internal Medicine, University Hospital Tuebingen, 72076 Tuebingen, Germany; Fabian.Riegg@med.uni-tuebingen.de (F.R.); Martina.Lutz@med.uni-tuebingen.de (M.S.L.); Bastian.Schmied@med.uni-tuebingen.de (B.J.S.); Jonas.Heitmann@med.uni-tuebingen.de (J.S.H.); Helmut.Salih@med.uni-tuebingen.de (H.R.S.); 2 DFG Cluster of Excellence 2180 “Image-Guided and Functional Instructed Tumor Therapy (iFIT)”, University of Tuebingen, 72076 Tuebingen, Germany; 3 Department I—General Pediatrics, Children’s Hospital, University Hospital Tübingen, Hematology/Oncology, 72076 Tuebingen, Germany; Manon.Queudeville@med.uni-tuebingen.de (M.Q.); peter.lang@med.uni-tuebingen.de (P.L.); 4 Department for Immunology, Eberhard Karls University, 72076 Tuebingen, Germany; gundram.jung@uni-tuebingen.de

**Keywords:** CD133, prominin-1, ADCC, NK cells, acute lymphoblastic leukemia, B-ALL, immunotherapy, Fc engineering

## Abstract

**Simple Summary:**

B cell acute lymphoblastic leukemia (B-ALL) is a common blood cancer characterized by proliferating and accumulating malignant, immature B cells within the body. Despite recent successes in B-ALL therapy, there is still a need for new therapeutic options. In the present study, we report on the characterization of 293C3-SDIE for the treatment of B-ALL. 293C3-SDIE is an improved anti-tumor antibody targeting CD133, a common protein on the surface of B-ALL cells. We demonstrated that 293C3-SDIE specifically induces activation of natural killer cells, which leads to lysis of B-ALL cells. Based on this study, we conclude that CD133 serves as a target for immune therapy, and treatment with 293C3-SDIE represents a promising therapeutic option in B-ALL therapy and warrants further preclinical and clinical evaluation.

**Abstract:**

In recent decades, antibody-dependent cellular cytotoxicity (ADCC)-inducing monoclonal antibodies (mAbs) have revolutionized cancer immunotherapy, and Fc engineering strategies have been utilized to further improve efficacy. A promising option is to enhance the affinity of an antibody’s Fc-part to the Fc-receptor CD16 by altering the amino acid sequence. Herein, we characterized an S239D/I332E-modified CD133 mAb termed 293C3-SDIE for treatment of B cell acute lymphoblastic leukemia (B-ALL). Flow cytometric analysis revealed CD133 expression on B-ALL cell lines and leukemic cells of 50% (14 of 28) B-ALL patients. 293C3-SDIE potently induced NK cell reactivity against the B-ALL cell lines SEM and RS4;11, as well as leukemic cells of B-ALL patients in a target antigen-dependent manner, as revealed by analysis of NK cell activation, degranulation, and cytotoxicity. Of note, CD133 expression did not correlate with BCR-ABL, CD19, CD20, or CD22, which are presently used as therapeutic targets in B-ALL, which revealed CD133 as an independent target for B-ALL treatment. Increased CD133 expression was also observed in MLL-AF4-rearranged B-ALL, indicating that 293C3-SDIE may constitute a particularly suitable treatment option in this hard-to-treat subpopulation. Taken together, our results identify 293C3-SDIE as a promising therapeutic agent for the treatment of B-ALL.

## 1. Introduction

In many cancer entities, the introduction of monoclonal antibodies (mAbs) has significantly improved the treatment options for patients. This is exemplified by the anti-CD20 mAb Rituximab and the anti-human epidermal growth factor receptor 2 (HER2)/neu mAbs Trastuzumab (Herceptin), the first clinically available antitumor antibodies, which have become a mainstay of therapy in patients with B cell non-Hodgkin’s lymphoma and HER2^+^ breast cancer, respectively [1,2]. Despite the advances achieved by the introduction of mAbs in cancer treatment, their success still has its limitations: Many patients do not respond, or respond for a limited time only. One major mechanism contributing to the therapeutic efficacy of mAbs is antibody-dependent cellular cytotoxicity (ADCC) [3]. In humans, ADCC is mainly mediated by natural killer (NK) cells after the interaction of surface Fc-receptors, e.g., CD16, and target cell surface-bound antibodies [4,5].

To enhance ADCC, one strategy is to modify Fc-parts in order to enhance their affinity to the Fc-receptor IIIa (FcRIIIa/CD16a). The amino acid substitutions S239D/I332E (SDIE) increase the Fc-part’s affinity to FcR in general, but with a more pronounced effect achieved for activating FcRIIIa/CD16a compared to the inhibitory FcRIIb/CD32b [6,7]. Glyco-engineering, to modify the Fc-part’s glycosylation pattern, represents another option to enhance immunostimulatory efficacy. Presently, glyco-optimized mAbs, such as the CD20 mAb Obinutuzumab, are approved for treatment of certain B cell malignancies, whereas many mAbs with amino acid substitutions in their Fc-part are currently being evaluated in clinical trials [8,9].

For the treatment of B cell acute lymphoblastic leukemia (B-ALL), ADCC-inducing mAbs are not the only immunotherapeutic strategy. Remarkable effects upon the treatment of relapsed/refractory (r/r) B-ALL have been observed by applying the CD19 × CD3 bispecific T cell engager (BiTE) Blinatumomab or anti-CD19 chimeric antigen receptor (CAR) T cell products such as Tisagenlecleucel [10,11]. Moreover, targeted cytotoxic drug delivery to CD22-expressing B-ALL cells by the antibody drug conjugate (ADC) Inotuzumab ozogamicin has been proven to be superior to standard therapy in r/r B-ALL [12]. However, the cure rate of only ~40% in adult ALL patients highlights the need for further therapeutic options [13]. To overcome antigen loss, a frequently seen escape mechanism in immunotherapy, current strategies aim to target multiple antigens, e.g., a dual CAR T cell therapy against CD19 and CD22, which is being tested in clinical trials (NCT03448393). Furthermore, the benefits and risks of CAR T cell therapy compared to Blinatumomab and Inotuzumab ozogamicin are also being addressed in clinical trials (NCT03628053).

During recent years, we introduced mAbs and antibody-related constructs carrying the SDIE modification for the immunotherapy of different leukemic and solid tumor entities [14,15,16,17]. Recently, we reported on the characterization of an Fc-optimized mAb, which targets FMS-like tyrosine kinase 3 (FLT3) expressed on the cell surface of leukemic cells in patients with acute leukemia [7,18]. Our construct, termed 4G8-SDIEM (FLYSYN), is undergoing clinical evaluation in a phase I study enrolling AML patients with the aim of eliminating minimal residual disease (NCT02789254). Besides FLYSYN, we also conceptualized an Fc-optimized mAb targeting the pentaspan transmembrane glycoprotein CD133 (prominin-1), which showed superior induction of NK cell reactivity in AML and in colorectal cancer (CRC) [19,20]. Beyond AML, CD133 has also been reported to be expressed in B-ALL [21]. Previous studies revealed that in B-ALL, CD133 expression is significantly correlated with a higher resistance to chemotherapy and is associated with a tendency toward poorer outcomes, a higher incidence of relapse, and death, as well as higher complete remission (CR) rates in CD133-negative ALL cases [22]. Moreover, previous studies revealed that CD133 is especially expressed in mixed-lineage leukemia (MLL)-AF4 ALL patients [23,24]. Nonetheless, the exact biological function of CD133 and its role in cancer remains to be fully elucidated [25].

Herein, we set out to characterize the Fc-optimized CD133 mAb 293C3-SDIE for targeting CD133 in the treatment of B-ALL with the aim of expanding the selection of mAb-targeted antigens for B-ALL patients, especially in the context of B-ALL subpopulations highly expressing CD133.

## 2. Materials and Methods

### 2.1. Antibody Production and Purification

293C3-SDIE and iso-SDIE were produced as described previously [19,20]. In brief, plasmids for heavy and light chains (in the case of iso-SDIE derived from MOPC21) were used to express antibodies in ExpiCHO cells (Gibco, Carlsbad, CA, USA) according to the manufacturer’s recommendations and purified by affinity (Mabselect; GE Healthcare, Chicago, IL, USA). This was followed by subsequent preparative size exclusion chromatography (HiLoad 16/60 Superdex 200; GE Healthcare, Chicago, IL, USA). Prior to use in functional experiments, the mAbs were cleared from endotoxins using the Endotrap HD kit from Hyglos (Bernried, Germany). Ultimately, the antibodies were run on analytical size exclusion columns (Superdex 200 Increase 10/300 GL; GE Healthcare; Chicago, IL, USA) and 4–12% gradient SDS-PAGE gels (Invitrogen; Carlsbad, CA, USA) using the gel filtration and Precision Plus standard from Bio-Rad (Hercules, CA, USA), respectively.

### 2.2. Cell Lines and Primary Material

The B-ALL cell lines REH, SEM, and RS4;11 were purchased from the German Collection of Microorganisms and Cell Cultures (Braunschweig, Germany). RS4;11 and REH were maintained in RPMI1640 (Gibco) and SEM in IMDM (Gibco). B-ALL cell lines were routinely tested for mycoplasma contamination and authenticated by flow cytometry-based immunophenotyping. Cells were cultured a maximum of one month prior to functional assays and regularly renewed from a frozen stock.

Peripheral blood samples of 28 patients with B-ALL at primary diagnosis were collected after informed consent in accordance with the Helsinki protocol and the local ethics committee (13/2007V, 819/2017B01). Peripheral blood mononuclear cells (PBMCs) and bone marrow cells of patients and healthy volunteers were isolated by density gradient centrifugation (Biocoll; Biochrom, Berlin, Germany) and viably stored in liquid nitrogen. One day prior to the functional assays, the PBMCs of healthy donors were cultured in RPMI1640 for 18–24 h. NK cells were purified from freshly isolated PBMCs of healthy volunteers using the Human NK Cell Isolation Kit (Miltenyi Biotec, Bergisch Gladbach, Germany) according to the manufacturer’s recommendations. Media were used with 10% heat-inactivated fetal calf serum (Biochrom) and 1% penicillin/streptomycin (Lonza, Basel, Switzerland), and cells were cultured at 37 °C and 5% CO_2_ in a humidified atmosphere.

### 2.3. Flow Cytometry

Flow cytometry was used to determine CD133 expression and 293C3-SDIE binding using the respective unlabeled mAbs, followed by species-specific PE conjugates. The cells were blocked with human or mouse IgG (Sigma-Aldrich, St. Louis, MO, USA) prior to the staining with mouse anti-human CD133 mAbs AC133, 293C3, W6B3C1 (Miltenyi Biotec), mouse anti-human CD22 (Biolegend, San Diego, CA, USA), 293C3-SDIE, or the corresponding isotype controls (BD Pharmingen, San Diego, CA, USA), followed by goat anti-mouse PE (Dako, Glostrup, Denmark) or donkey anti-human PE (Jackson ImmunoResearch, West Grove, PA, USA). To identify different cellular subsets, PBMCs or bone marrow cells were additionally counterstained with fluorescent-labeled CD19-FITC, CD45-PeCy7 (all BD Pharmingen), CD10-PE-Cy7, CD20-BV510, CD56-APC, CD3-APC/Fire750, CD14-BV785, HLA-DR-BV650, or CD34-APC (all Biolegend) mAbs. The expression levels of CD20, CD19, CD34, and CD10 from the PBMCs of B-ALL patients were obtained by the flow cytometry diagnostic laboratory of University Hospital Tuebingen according to standard procedures at diagnosis. CD107a-PE and CD69-PE (both BD Pharmingen) were used for studies on NK cell activation and degranulation. Dead cells were excluded with 7-AAD (Biolegend) staining. FACS Canto II and FACS Fortessa (both BD Biosciences; Heidelberg, Germany) devices were used for data acquisition, followed by data analysis with FlowJo software (FlowJo LCC, Ashland, OR, USA). Specific fluorescence intensity (SFI) levels were calculated by dividing the mean fluorescence intensity (MFI) of the measured antigen by the MFI of the respective isotype control. Surface expression was considered positive in case of ≥20% and SFI ≥ 1.5.

### 2.4. Analysis of NK Cell Activation and Degranulation

The PBMCs of healthy donors were co-cultured with or without SEM and RS4;11 or primary B-ALL cells at an effector to target (E:T) ratio of 2.5:1 and in the presence or absence of 293C3-SDIE/iso-SDIE (1 µg/mL). Prior to co-cultivation, target cells were loaded with 1 μM CellTrace™ violet cell proliferation dye (Thermo Fisher Scientific, Waltham, MA, USA). NK cells were identified as CD56^+^ CD3^–^ lymphocytes (Figure S2) and NK cell activation was determined by CD69 expression, measured after 24 h by flow cytometry analysis. NK cell degranulation was analyzed by flow cytometry after culturing the cells for 4 h in the presence of CD107a-PE, GolgiStop, and GolgiPlug (BD Biosciences).

### 2.5. Analysis of NK Cell Cytotoxicity

Lysis of leukemic cells in the presence or absence of 293C3-SDIE/iso-SDIE (1 µg/mL) was determined by 2 h Europium cytotoxicity assays as previously described [15]. The specific lysis rate was calculated as follows: 100 × (experimental release − spontaneous release)/(maximum release − spontaneous release). Unless otherwise stated, the lysis rates are shown as means of technical triplicates with standard deviation.

### 2.6. Statistics

GraphPad Prism 8 (GraphPad Software, San Diego, CA, USA) was used for statistical analysis. Data are displayed as mean ± SEM and boxplots as median with 25% and 75% percentile and min. to max. whiskers. Normal distribution was tested with the Shapiro–Wilk test. The 95% confidence level was utilized. For correlation analyses of B-ALL surface makers, Pearson’s correlation was used, while in the case of BCR-ABL, MLL-AF4, CR, and one-year survival rate, an unpaired *t*-test was performed, and in the case of ALL risk profiles, a Kruskal–Wallis test and subsequent Dunn’s multiple comparisons tests were calculated. With regard to functional data, *p*-values were calculated by one-way ANOVA, followed by Tukey’s multiple comparison tests. Statistically significant *p*-values (*p* < 0.05) are marked by “*”, whereas non-significant *p*-values are marked by “ns”.

## 3. Results

### 3.1. Characterization of Fc-Optimized 293C3-SDIE on B-ALL Cells

**Figure 1:**
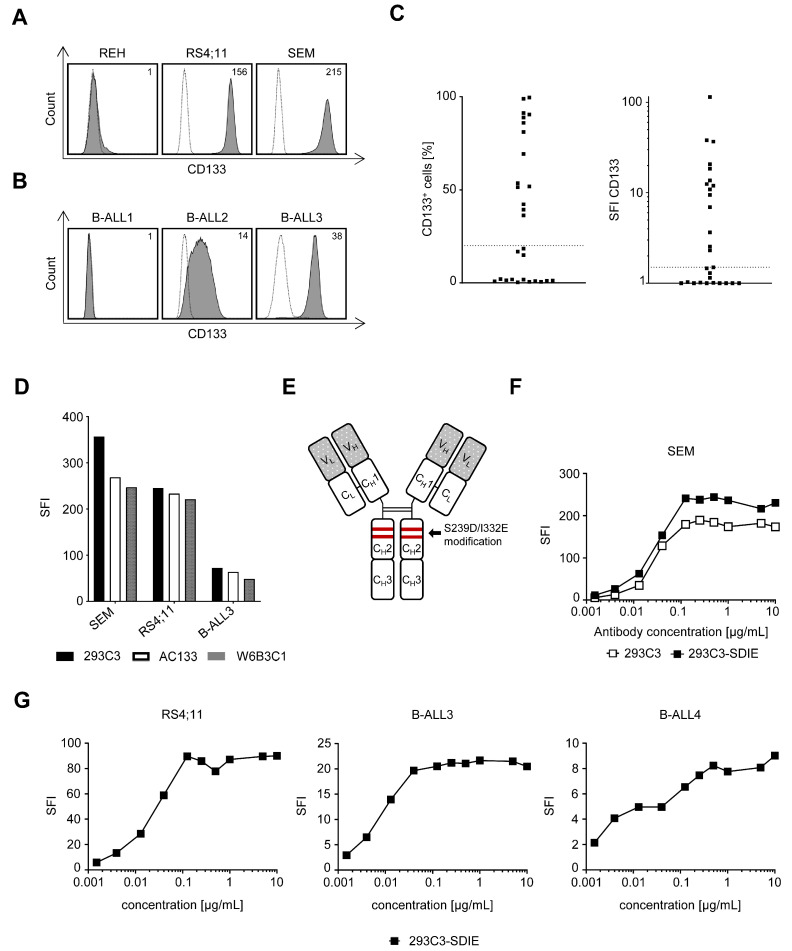
Characterization of 293C3-SDIE in B cell acute lymphoblastic leukemia (B-ALL). (**A**–**C**) B-ALL cell lines and primary leukemic cells of B-ALL patients were incubated with anti-human CD133 antibody clone 293C3 or mIgG2b as isotype control (both 10 µg/mL) and analyzed by flow cytometry. (**A**,**B**) Exemplary data of CD133 expression on the B-ALL cell lines REH, SEM, and RS4;11 and three B-ALL patients (B-ALL1–3) (shaded peaks, anti-CD133; open peaks, control). Specific fluorescence intensity (SFIs) levels are depicted in the upper right corner. (**C**) Pooled data of CD133 expression on leukemic cells of primary B-ALL patients (*n* = 28) depicted as % CD133^+^ B-ALL blasts (left, dotted line: 20% surface expression) and SFI levels (right, dotted line: SFI = 1.5). (**D**) The B-ALL cell lines SEM and RS4;11, as well as the leukemic cells of an exemplary B-ALL patient (B-ALL3), were incubated with mouse anti-human CD133 antibody clones 293C3, AC133, W6B3C1, or mIgG1 and mIgG2b as isotype controls (all 1 µg/mL) and analyzed by flow cytometry. (**E**) Schematic illustration of 293C3-SDIE. (**F**) The B-ALL cell line SEM was incubated with increasing concentrations of the mouse anti-human CD133 antibody 293C3 or 293C3-SDIE and mIgG2b or iso-SDIE as isotype controls (10 µg/mL) and analyzed by flow cytometry. (**G**) The B-ALL cell line RS4;11 and the leukemic cells of two exemplary B-ALL patients (B-ALL3 and B-ALL4) were incubated with increasing concentrations of 293C3-SDIE or iso-SDIE (10 µg/mL) and analyzed by flow cytometry.

We studied the specific binding of the CD133 antibody clone 293C3 to determine the surface expression of CD133 on the B-ALL cell lines REH, RS4;11, and SEM, primary leukemic cells of 28 patients diagnosed with B-ALL, as well as on PBMCs and bone marrow cells of healthy donors. Flow cytometric analysis revealed high expression on RS4;11 and SEM, whereas the REH cells were negative for CD133 (Figure 1A), which is in line with previously reported results for other CD133 mAb clones [23]. Furthermore, the screening of 28 B-ALL patients showed substantial expression (≥20%; SFI ≥ 1.5) of CD133 on leukemic cells in 50% (14 of 28) with SFI levels up to 114.9 in the analyzed patient samples (Figure 1B,C). Besides that, no CD133 expression was found on the PBMC subsets, while minor low expression was observed on healthy CD34^+^ bone marrow cells (Figure S1). Since different CD133 clones have been used in previous studies of AML and CRC [19,20], revealing different CD133 binding patterns, we compared the most common clones AC133, W6B3C1, and 293C3 in B-ALL. Comparative flow cytometry analysis of B-ALL cells for specific binding of the CD133 clones AC133, 293C3, and W6B3C1 revealed no remarkable differences in binding using the different CD133 clones (Figure 1D). This confirmed the suitability of our construct containing the clone 293C3, termed 293C3-SDIE, for targeting B-ALL. As illustrated in Figure 1E, 293C3-SDIE is a chimerized and Fc-optimized antibody (S293D/I332E modification). Dose titration experiments revealed that 293C3-SDIE showed no binding differences compared to the mouse 293C3 antibody on SEM cells (Figure 1F). Dose titration experiments of 293C3-SDIE using B-ALL cell lines and primary B-ALL cells further revealed that 1 µg/mL was sufficient for saturating antigen binding (Figure 1G). The characteristics of the B-ALL cell lines and primary B-ALL cells used in the functional experiments are depicted in Table S1.

### 3.2. Induction of NK Cell Reactivity against B-ALL Cell Lines

**Figure 2:**
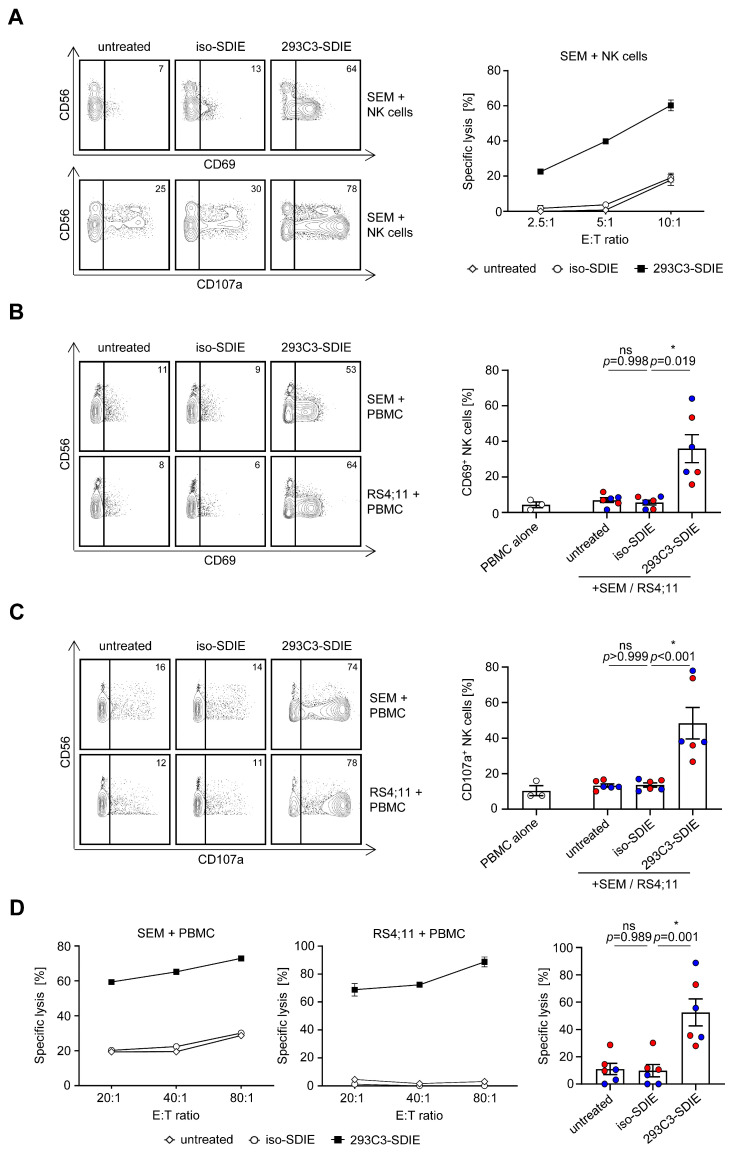
Induction of natural killer (NK) cell reactivity against CD133^+^ B-ALL cell lines. (**A**–**D**) The B-ALL cell lines SEM and RS4;11 were co-cultured with purified NK cells or PBMCs of healthy donors (effector to target (E:T) ratio of 2.5:1 or indicated E:T ratio) in the presence or absence of 293C3-SDIE and iso-SDIE (both 1 µg/mL) for 24 h (activation), 4 h (degranulation), or 2 h (Europium assay). To determine the NK cell activation and degranulation, the NK cells were identified as CD56^+^CD3^−^ lymphocytes and stained with CD69 or CD107a with subsequent flow cytometric analysis. Target cell lysis of different B-ALL cell lines was analyzed by Europium assays. (**A**) Exemplary data of purified NK cells tested against the B-ALL cell line SEM. (**B**,**C**) Exemplary data from one PBMC donor (left panel) and pooled results of three PBMC donors tested with SEM (red) and RS4;11 (blue) (right panel) are shown (mean ± SEM). (**D**) Exemplary data from one PBMC donor (left panel) and pooled results of three PBMC donors tested with SEM (red) and RS4;11 (blue) at an E:T ratio of 80:1 (right panel) are shown (mean ± SEM). ns, not significant; * significant (*p* < 0.05).

Next, we analyzed whether and how 293C3-SDIE induces NK cell reactivity and target-specific lysis of B-ALL cell lines. Therefore, we co-cultured the purified NK cells or PBMCs of healthy donors with SEM and RS4;11 cells as target cells in the presence or absence of 293C3-SDIE and iso-SDIE as the control. Flow cytometric analysis of CD69 expression on the NK cells showed significant induction of NK cell activation by 293C3-SDIE, whereas in the presence of iso-SDIE, no effects were observed (Figure 2A,B). This increase in NK cell activity induced by the presence of 293C3-SDIE was mirrored by a significant induction of NK cell degranulation, as revealed by flow cytometric analysis of CD107a expression (Figure 2A,C). Finally, Europium-based cytotoxicity assays confirmed that treatment with 293C3-SDIE, compared to the isotype control, resulted in induction of target-antigen restricted lysis of the B-ALL cell lines (Figure 2A,D). Of note, the analyses using purified NK cells compared to PBMCs showed similar results for 293C3-SDIE treatment.

### 3.3. Induction of NK Cell Reactivity against Primary B-ALL Cells

**Figure 3:**
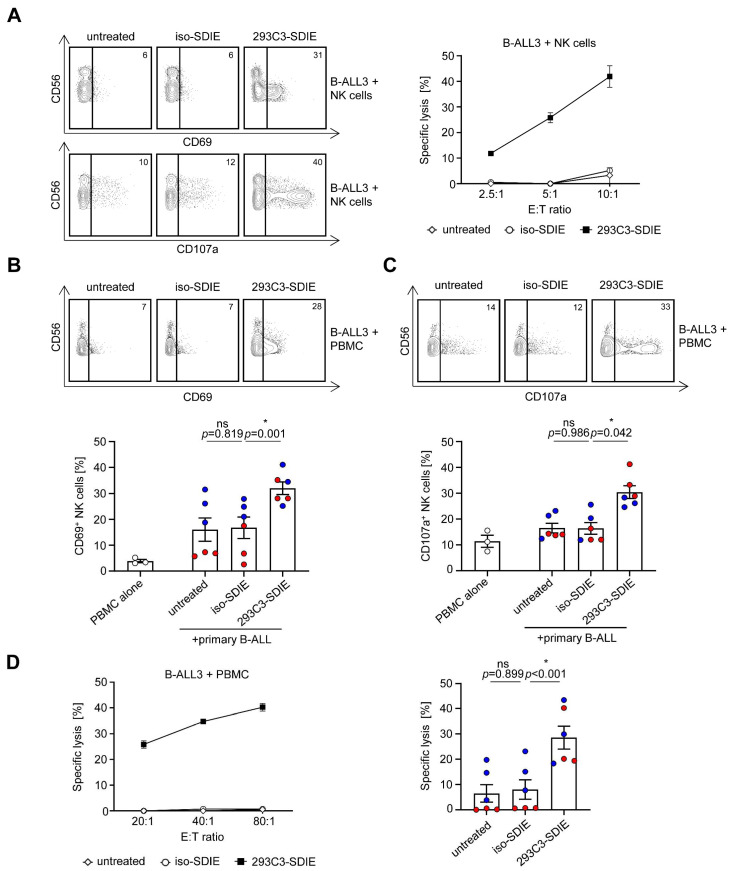
Induction of NK cell reactivity against CD133^+^ primary B-ALL cells. (**A**–**D**) Cells of CD133^+^ B-ALL patients were co-cultured with purified NK cells or PBMCs of healthy donors (E:T ratio of 2.5:1 or indicated E:T ratio) in the presence or absence of 293C3-SDIE and iso-SDIE (both 1 µg/mL) for 24 h (activation), 4 h (degranulation), or 2 h (Europium assay). To determine the NK cell activation and degranulation, the NK cells were identified as CD56^+^CD3^−^ lymphocytes and stained with CD69 or CD107a with subsequent flow cytometric analysis. Target cell lysis of different B-ALL cell lines was analyzed by Europium assays. (**A**) Exemplary data of purified NK cells tested against primary B-ALL cells (B-ALL3). (**B**,**C**) Exemplary data from one PBMC donor (upper panel) and pooled results of three PBMC donors tested with two B-ALL patients (B-ALL3: red; B-ALL4: blue) (lower panel) are shown (mean ± SEM). (**D**) Exemplary data from one PBMC donor (left panel) and pooled results of three PBMC donors tested with two B-ALL patients (B-ALL3: red; B-ALL4: blue) at an E:T ratio of 80:1 (right panel) are shown (mean ± SEM). ns, not significant; * significant (*p* < 0.05).

Next, we determined whether the induction of NK cell reactivity by 293C3-SDIE observed with B-ALL cell lines as target cells was also mirrored when primary B-ALL cells were employed in functional analyses. Again, the purified NK cells or PBMCs of healthy donors were used as effector cells in co-cultures with primary B-ALL cells in the presence or absence of 293C3-SDIE and iso-SDIE as the control. Flow cytometric analysis of CD69 and CD107a expression revealed profound induction of NK cell activation and degranulation in the presence of 293C3-SDIE, while no effects were observed in the presence of iso-SDIE (Figure 3A–C). Finally, target cell-restricted lysis of primary B-ALL cells by 293C3-SDIE was confirmed by using Europium-based cytotoxicity assays. Primary B-ALL cells, including cells of an MLL-AF4^+^ B-ALL patient, were effectively killed by the PBMCs of healthy donors using 293C3-SDIE, while treatment with iso-SDIE induced no lysis of leukemic cells (Figure 3A,D). Again, treatment of primary B-ALL cells with 293C3-SDIE showed comparable results by using purified NK cells or PBMCs.

### 3.4. Clinical Characteristics of Primary B-ALL Patients and Prognostic Evaluation of CD133 Expression

**Figure 4:**
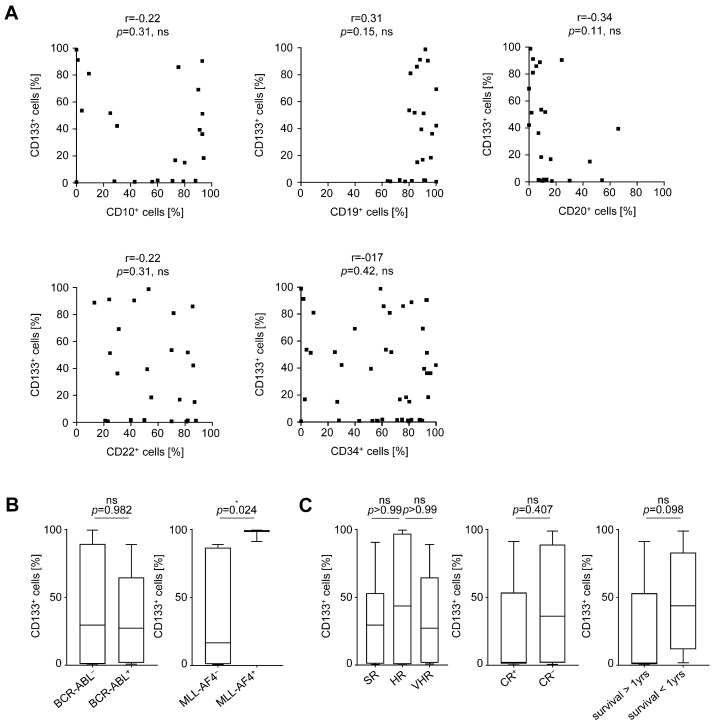
Association of CD133 expression with B-ALL-related antigens and clinical data. (**A**–**C**) Primary leukemic cells of B-ALL patients were incubated with anti-human CD133 antibody clone 293C3 or mIgG2b as isotype control (both 10 µg/mL) and analyzed by flow cytometry. (**A**) Association of CD133 expression with the B-ALL antigens CD10, CD19, CD20, CD22, CD34, and clinical data. (**B**) BCR-ABL positivity and MLL-AF4 positivity; (**C**) risk stratification according to GMALL, complete response, and one-year-survival on primary B-ALL cells. *P*, *p*-value; r, Pearson’s correlation coefficient; SR, standard risk; HR, high risk; VHR, very high risk; CR, complete remission; ns, not significant; * significant (*p* < 0.05).

We next comparatively analyzed the expression of CD133 and other commonly described B-ALL targets related to disease pathophysiology and/or serving for therapeutic approaches on leukemic cells in our B-ALL patient cohort (Table 1), and we analyzed the potential correlations (Figure 4A). No clear correlation of CD133 with CD10, CD19, CD20, CD22, or CD34 positivity was observed. Analysis of genetic aberrations such as BCR-ABL translocation showed no association with CD133 expression; patients with MLL rearrangement presented with significantly high levels of surface CD133 on their leukemic cells (Figure 4B). Interestingly, we observed no clear correlation of CD133^+^ blasts in B-ALL patients with a higher risk profile compared to standard risk, indicating that 293C3-SDIE could be used independently of risk association in B-ALL patients. However, patients exhibiting higher CD133 surface expression more often did not reach CR upon treatment. Furthermore, the one-year-survival rate was higher in B-ALL patients with lower CD133 surface expression (Figure 4C and Figure S3).

## 4. Discussion

In this study, we reported on the preclinical characterization of 293C3-SDIE, a chimerized and Fc-optimized mAb targeting CD133 for the treatment of B-ALL. Beyond other Fc-optimized antibodies directed to other target antigens (e.g., [14,15,16,17]), we recently evaluated 293C3-SDIE for treatment of AML and CRC [19,20]. Based on these results, an additional evaluation in B-ALL appeared warranted, since CD133 is reportedly expressed in 30–40% of ALL cases and constitutes a promising target antigen for B-ALL therapy [22,26,27]. After validating the CD133 expression in our patient cohort, which was found positive in 50% of cases, and after analyzing the binding characteristics of 293C3-SDIE on B-ALL cells, we conducted multiple experiments to demonstrate that 293C3-SDIE potently induces NK cell reactivity against B-ALL cell lines and primary B-ALL samples.

Recently, various immunotherapies were FDA-approved for ALL: Blinatumomab, a CD19 × CD3 bispecific mAb, Inotuzumab ozogamicin, a CD22-targeting ADC, and Tisagenlecleucel, an anti-CD19 CAR T cell product, have remarkably improved the outcome of r/r B-ALL patients [10,11,12,28]. In addition, Rituximab is successfully used “off-label” in combination with chemotherapy in CD20^+^ B-ALL patients (~30%). Beyond immunological treatment options, tyrosine kinase inhibitors such as Imatinib, Dasatinib, and Ponatinib have become part of or are being evaluated for the treatment of BCR-ABL^+^ patients [29,30]. However, the overall cure rate of only ~40% in adult ALL patients highlights the need to develop additional new therapeutic options [13]. Particularly, expanding the repertoire of antigens targeted by immunotherapy approaches appears promising, since antigen loss is a major escape mechanism during immunotherapy. Downmodulation and genetic mutation of target genes, or cell lineage change with antigen loss, constitute the involved mechanisms, which were partly reported upon treatment with Inotuzumab ozogamicin, Blinatumomab, and Tisagenlecleucel [13,31,32,33]. The introduction of CD133 as an additional target thus appears promising, especially since CD133 expression is not associated with the prevalence of other therapeutic targets such as BCR-ABL, CD19, CD20, and CD22. In addition, compared to T cell immunotherapeutics or ADC, mAbs such as 293C3-SDIE are expected to cause lower toxicity and side effects, which underlines their suitability for therapeutic approaches targeting multiple antigens.

As illustrated by the success of Rituximab in lymphoma treatment, ADCC plays a crucial role for efficacy of antitumor mAbs, especially in hematological malignancies [5,34]. Therefore, developing new strategies to increase the ADCC efficacy of antitumor mAbs, which is mainly mediated by the stimulation of NK cells, is of great interest. A promising approach is Fc engineering, which comprises, amongst others, the optimization of ADCC by enhancing the affinity to Fc-receptor IIIa (FcRIIIa/CD16a). One way to achieve this is altering the Fc-part’s glycosylation pattern. This is exemplified by Obinutuzumab, a glyco-optimized CD20 antibody, approved for the treatment of chronic lymphocytic leukemia, showing superior treatment results compared to Rituximab [9]. Furthermore, changing the amino acid sequence, exemplified by S239D/I332E (SDIE) in 293C3-SDIE, represents another option. Interestingly, the SDIE-modified CD19 antibody Tafasitamab was recently FDA-approved for the treatment of r/r diffuse large B cell lymphoma (DLBCL), and several other Fc-optimized antibodies carrying the SDIE modification are currently being evaluated in clinical trials [35,36].

While several studies have shown no significant association of CD133^+^ expression with age, gender, or clinical characteristics, e.g., percentage of leukemic blast cells in peripheral blood/bone marrow of ALL patients, CD133^+^ ALL cells have shown a higher degree of immaturity [22]. Additionally, CD133 expression has been shown to be significantly correlated with a higher resistance to chemotherapeutic treatment and is associated with a tendency toward poorer outcomes, a higher incidence of relapse, and death, and vice versa, higher CR rates in CD133-negative ALL cases [22]. This is in line with our results obtained by flow cytometric analysis using mAbs 293C3, which showed a tendency toward higher CD133 expression in patients not reaching CR and with lower one-year survival rates. These findings, taken together, also support the rationale for targeting CD133 in B-ALL. Worth mentioning is the frequently observed overexpression of CD133 in MLL-AF4-rearranged B-ALL patients, which is in line with the results of recent studies that revealed that PROM1, the corresponding CD133 gene, is a direct target of MLL-AF4 gene regulation [23]. Interestingly, further studies have shown that PROM1/CD133 is essential for the survival of MLL-AF4-dependent ALL cells [23,24]. Taken together, this underlies the potential of a CD133-targeted therapy, particularly in MLL-AF4 ALL, which accounts for 5–10% of all B-ALL patients that have a particularly poor prognosis [23,37,38]. Especially in childhood B-ALL, infants with MLL-AF4 B-ALL have a very poor prognosis [39]. In our study, we showed that 293C3-SDIE allows for the potent induction of NK cell ADCC against MLL-AF4-positive cell lines, as well as against primary leukemic cells of MLL-AF4-rearranged B-ALL patients. Notably, potent ADCC was also observed in patients with low CD20 expression, which further underlines the potential of 293C3-SDIE. However, to ultimately determine the efficacy and appropriate patient subpopulation, further characterization of 293C3-SDIE in extended B-ALL patient populations is needed. Potentially, 293C3-SDIE could be combined with chemotherapy or other immunotherapies. In a potential subsequent clinical study, 293C3-SDIE could be tested in a minimal residual disease situation, similar to the phase I study with our construct 4G8-SDIEM (FLYSYN), being tested against AML patients (NCT02789254).

Apart from 293C3-SDIE, other therapeutics directed at CD133 are presently undergoing pre-/clinical development. These comprise aptamers, bi-/tri-/tetraspecific mAbs, bi-/tri-/tetraspecific killer engager (Bi-/Tri-/TetraKE), CAR T cells, dendritic cell (DC)-based vaccination strategies, immunotoxins, and nanoparticles [25,40]. Particularly CD133 CAR T cell products have partly reached the stage of clinical evaluation [41,42]. Compared to CD133 CAR T cells, 293C3-SDIE shows several advantages, including a quicker applicability, as the manufacturing time of three to four weeks in the case of CAR T cells would not be required, and it would come at lower costs. Even more so since our previous studies showed that 293C3-SDIE can be efficiently produced with only a minor aggregation tendency, which overcomes the issues occurring when producing more artificial compounds such Bi-/Tri-/TetraKEs [19,20]. In the case of immunotoxins, a high internalization rate of the target antigen is desirable, as shown with CD22 upon the highly effective treatment with Inotuzumab ozogamicin in B-ALL [12]. In the case of targeting CD133 with constructs comprising the mAb clone 293C3, we observed a relatively low antigen shift, which promised surface-expressed CD133 as an optimal target for NK cell therapy [19].

A crucial aspect in the characterization of tumor mAbs is evaluating the potential toxicity, especially in case of non-tumor-exclusive antigens such has CD133, which is expressed, amongst others, on healthy hematopoietic progenitor cells. This was addressed in our previous studies with 293C3-SDIE, which revealed no relevant in vitro toxicity against hematopoietic progenitor cells, most likely due to—compared to leukemic cells—lower CD133 antigen levels [19]. In line, two clinical phase I studies and one clinical phase II study, using anti-CD133 CAR T cells/dendritic cells, revealed no overt “on-target off-tumor” side effects, especially no intolerable toxicity against healthy hematopoietic progenitor cells [41,42,43]. Taken together, the currently assumed favorable toxicity profile of 293C3-SDIE and CD133-targeting mAbs in general prompts the application in elderly and frail patients that are currently not eligible for high-dose chemotherapy or CAR T cell products. Accordingly, further preclinical and clinical studies are warranted to determine the suitability of 293C3-SDIE for B-ALL therapy.

## 5. Conclusions

Herein, we reported on the preclinical characterization of 293C3-SDIE, a chimerized and Fc-optimized mAb targeting CD133, for the treatment of B-ALL. Treatment with 293C3-SDIE potently induced NK cell reactivity against B-ALL cells in a target antigen-dependent manner; thus, we conclude that 293C3-SDIE constitutes a promising immunotherapeutic in B-ALL therapy.

## Figures and Tables

**Table 1:** Patients characteristics.

Characteristics	Number of Patients (%) (*n* = 28)
Sex	
Male	16 (57)
Female	12 (43)
Median age (years)	40 (range 0–81)
B-ALL type	
Pro-B-ALL	5 (18)
Common ALL	18 (64)
Pre-B-ALL	5 (18)
BCR-ABL status	
positive	12 (43)
negative	16 (57)
MLL-AF4 status	
positive	3 (11)
negative	6 (21)
NA	19 (68)
Blood count	
WBC (G/L)	84 (range 4.7–463)
Hb (g/dL)	9.6 (range 4.8–13.6)
Plt (G/L)	80 (range 11–280)
Risk stratification *	
SR	6 (21)
HR	9 (32)
VHR	12 (43)
NA	1 (4)
Complete remission ^π^	15 (54)

NA, not available; WBC, white blood count; Hb, hemoglobin; plt, thrombocytes; SR, standard risk; HR, high risk; VHR, very high risk; * according to GMALL 07/2003; ^π^ best response observed during first-line therapy.

## Data Availability

The data presented in this study are available on request from the corresponding author. The data are not publicly available due to the used patient material.

## Supplementary Materials

The following are available online at https://www.mdpi.com/article/10.3390/cancers13071632/s1, Figure S1. CD133 expression on healthy cells. Figure S2. Gating strategy identifying NK cells. Figure S3. Association of CD133 expression (SFI) with clinical data. Table S1. Biological characteristics of employed B-ALL cells.
