# Supplementary material for: An Fc-Optimized CD133 Antibody for Induction of NK Cell Reactivity against B Cell Acute Lymphoblastic Leukemia
Source: Cancers (Basel). 2021 Apr 1;13(7):1632. doi: 10.3390/cancers13071632 (PMC8036612; doi:10.3390/cancers13071632)
Supplement: Supplementary file 1 [file cancers-13-01632-s001.pdf]

cancers MDPI

Supplementary Materials: An Fc-optimized CD133 Antibody for Induction of NK Cell Reactivity against B Cell Acute Lymphoblastic Leukemia

Fabian Riegg, Martina S. Lutz, Bastian J. Schmied, Jonas S. Heitmann, Manon Queudeville, Peter Lang, Gundram Jung, Helmut R. Salih and Melanie Märklin

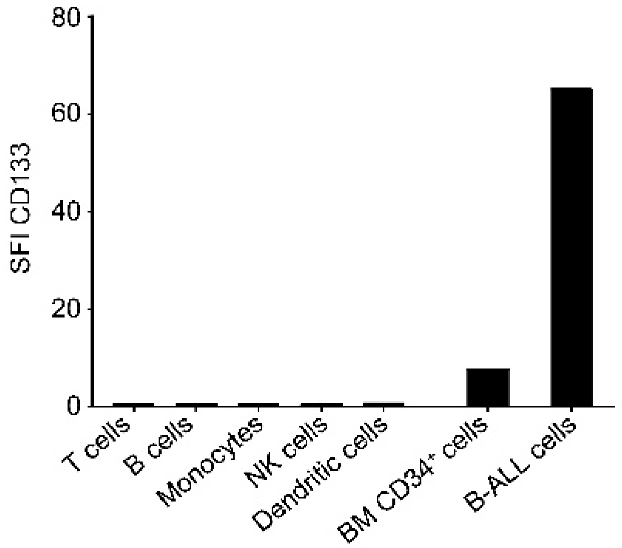

Figure S1. CD133 expression on healthy cells. PBMCs and bone marrow cells of healthy donors and primary leukemic cells of one B-ALL patient (B-ALL3) were incubated with anti-human CD133 antibody clone 293C3 or mIgG2b as isotype control (both 1 µg/ml) and analyzed by flow cytometry. BM, bone marrow; SFI, specific fluorescence intensity.

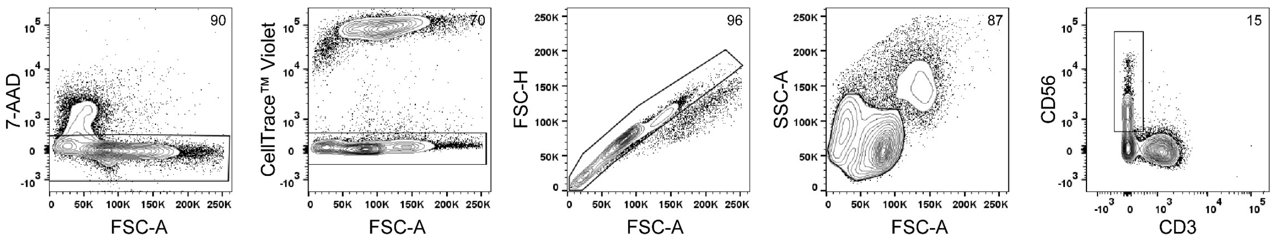

Figure S2. Gating strategy identifying NK cells. In Figure 2A–C/3A–C, B-ALL cells were co-cultured with the purified NK cells or PBMCs of healthy donors (E:T of 2.5:1) in the presence or absence of 293C3-SDIE and iso-SDIE (both 1 µg/mL) for 24 h (activation) and 4 h (degranulation). Prior to co-cultivation, target cells were loaded with CellTrace™ Violet cell proliferation dye. The depicted flow charts show the exemplary gating strategy to identify CD56+CD3 lymphocytes (NK cells) within PBMC. FSC, forward scatter; A, area; H, height.

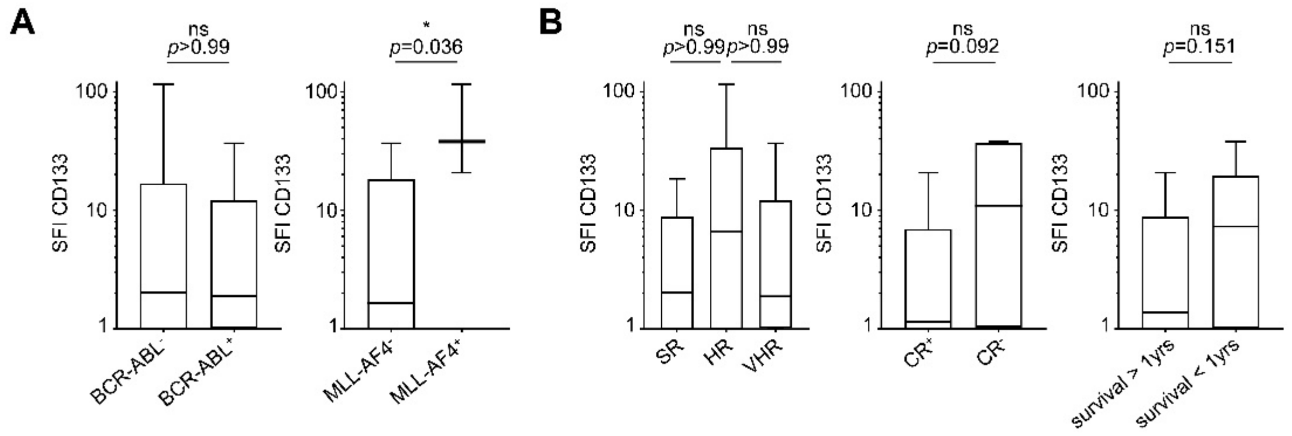

Figure S3. Association of CD133 expression (SFI) with clinical data. (**A**,**B**) Primary leukemic cells of B-ALL patients were incubated with anti-human CD133 anti-body clone 293C3 or mIgG2b as isotype control (both 10 µg/mL) and analyzed by flow cytometry. (A) BCR-ABL positivity and MLL-AF4 positivity; (B) risk stratification according to GMALL, complete response, and one-year survival on primary B-ALL cells. *p*, *p*-value; r, Pearson's correlation coefficient; SR, standard risk; HR, high risk; VHR, very high risk; CR, complete remission; SFI, specific fluorescence intensity.

Table 1. Biological characteristics of employed B-ALL cells.

Cell line/ B-ALL patient	B-ALL type[1]	Age [1]	Sex [1]	Risk	BCR-ABL[1,2]	MLL-AF4[1,2]	% CD133	% CD20
SEM	n.a.	5	f	n.a.	-	+	100	n.a.
RS4;11	n.a.	32	f	n.a.	-	+	100	n.a.
B-ALL3	pro B	63	m	HR	-	+	98.8	1
B-ALL4	common	50	f	VHR	+	-	88.8	8

[1] Information on the biological characteristics of the cell lines were derived from German Collection of Microorganisms and Cell Cultures. [2] B-ALL patients were assessed by PCR or FISH.; n.a., not available; f, female; m, male; BCR, breakpoint cluster region; ABL, Abelson murine leukemia viral oncogene homolog 1; -, negative; +, positive; HR, high risk; VHR, very high risk; MLL, mixed-lineage leukemia.
